# Focus on Hypoxia-Related Pathways in Pediatric Osteosarcomas and Their Druggability

**DOI:** 10.3390/cells9091998

**Published:** 2020-08-31

**Authors:** Marina Pierrevelcin, Quentin Fuchs, Benoit Lhermitte, Melissa Messé, Eric Guérin, Noelle Weingertner, Sophie Martin, Isabelle Lelong-Rebel, Charlotte Nazon, Monique Dontenwill, Natacha Entz-Werlé

**Affiliations:** 1 Laboratory of Bioimaging and Pathologies, UMR CNRS 7021, 67405 Illkirch, France; marina.pierrevelcin@etu.unistra.fr (M.P.); quentin.fuchs@etu.unistra.fr (Q.F.); Benoit.lhermitte@chru-strasbourg.fr (B.L.); melissa.messe@etu.unistra.fr (M.M.); sophie.martin@unistra.fr (S.M.); isabelle.lelong-rebel@unistra.fr (I.L.-R.); monique.dontenwill@unistra.fr (M.D.); 2 Pathology Department, University Hospital of Strasbourg, 67098 Strasbourg, France; Noelle.weingertner@chru-strasbourg.fr; 3 Oncobiology, Laboratory of Biochemistry and Molecular Biology, University Hospital of Strasbourg, 67098 Strasbourg, France; Eric.guerin@chru-strasbourg.fr; 4 Pediatric Oncohematology Unit, University Hospital of Strasbourg, 67098 Strasbourg, France; Charlotte.nazon@chru-strasbourg.fr

**Keywords:** osteosarcoma, hypoxia, progression, immunity, druggability, preclinical models

## Abstract

Osteosarcoma is the most frequent primary bone tumor diagnosed during adolescence and young adulthood. It is associated with the worst outcomes in the case of poor response to chemotherapy and in metastatic disease. While no molecular biomarkers are clearly and currently associated with those worse situations, the study of pathways involved in the high level of tumor necrosis and in the immune/metabolic intra-tumor environment seems to be a way to understand these resistant and progressive osteosarcomas. In this review, we provide an updated overview of the role of hypoxia in osteosarcoma oncogenesis, progression and during treatment. We describe the role of normoxic/hypoxic environment in normal tissues, bones and osteosarcomas to understand their role and to estimate their druggability. We focus particularly on the role of intra-tumor hypoxia in osteosarcoma cell resistance to treatments and its impact in its endogenous immune component. Together, these previously published observations conduct us to present potential perspectives on the use of therapies targeting hypoxia pathways. These therapies could afford new treatment approaches in this bone cancer. Nevertheless, to study the osteosarcoma cell druggability, we now need specific in vitro models closely mimicking the tumor, its intra-tumor hypoxia and the immune microenvironment to more accurately predict treatment efficacy and be complementary to mouse models.

## 1. Introduction

The overall survival (OS) of osteosarcoma (OTS) patients has remained stable for three decades. For that reason, it is important to find new therapeutic strategies and new biomarkers to be able to predict the outcome and refine the prognosis of those children from the diagnosis. Pediatric high-grade OTS is the most common primary malignant bone tumor in children and adolescents. Those tumors account for more than 50% of primary bone cancers each year. The annual incidence is around 2 per million persons [1,2] with an incidence peak during puberty in a gender-dependent manner (males are more affected than females) [3]. Ten-year OS is 65% for patients diagnosed without metastases and decreases to 20% for patients with metastatic disease at diagnosis [4]. The most frequent tumor localizations are metaphyseal areas of long bones (e.g., 42% in the femur, 19% in the tibia and 10% in humerus) [5]. These metaphyseal bones are close to the growth plates, where there is rapid bone growth. OTS is characterized by an impaired balance between the osteoblast, osteoclast and the mesenchymal precursor activities. They are defined by an extracellular osteoid and sometimes chondroid matrix produced by the malignant osteoblasts and a rapid proliferation with early micro- and macro-metastases [5]. At diagnosis, 80% of the patients present “invisible” micro-metastasis and 23% of the patients will have already visible metastases, which are located for more than two-thirds of the cases in the lungs and 16% in bones [4,5].

**Figure 1:**
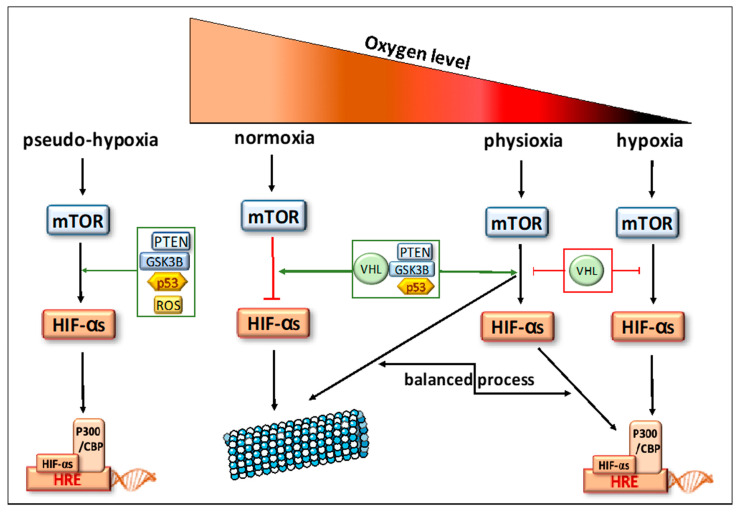
Oxygen tensions in bone tissues. Description of the signaling pathways controlling responses to oxygen variations.

This bone cancer is known as a highly necrotic tumor even at diagnosis, where this histological feature is frequently observed on biopsies. This necrosis might be the consequence of the excessive and rapid growth of cancer cells, which are proficient in creating a hypoxic microenvironment [6,7] and an abnormal neoangiogenesis [8,9]. Besides finding tumor necrosis at biopsy, OTS is also characterized by necrotic rate assessment on surgical tumor resection after a neoadjuvant multichemotherapy approach. The histological Huvos grading classifies OTS patients in 2 groups considering the number of residual tumor cells and their paired necrotic percentage [7,10]. A rate of less than 10% viable cells is the witness of good histological response (GR): grade III GRs have less than 10% residual tumor cells and grade IV GRs have a complete tumor necrosis. A rate above 10% viable cells is related to a poor histological response (PR): grade II PR is between 10 and 50% residual tumor cells and grade I PR is above 50% of residual tumor cells. The 5-year OS in localized GR patients is 80%, whereas this OS decreases dramatically at less than 30% in patients with PR, an unresectable tumor, a primitive metastatic tumor or a therapeutic resistance [7,11]. Tumor necrosis is believed to represent the endpoint of severe chronic hypoxia. This necrosis can promote oxidative stress and modify metabolic responses due to inducing hypoxia-related biomarkers. Hypoxia itself can induce necrosis in the heart of the tumor, but, oppositely, can also induce inhibition of apoptosis and necrosis during cancer progression and treatment [12]. The cancer-related hypoxia is in addition to in vivo physiological oxygen tension, called physioxia (Figure 1). The atmospheric oxygen level (21% oxygen) is a non-physiological environment and can be considered as hyperoxia. The physioxia within human organs ranges from 5 to 9% depending on body regions (e.g., breast is at 8.5%, bone marrow at 7% and 5% for bones) [12,13,14]. Therefore, the hypoxic level can be probably defined in tumors and OTS as the rate of oxygen, which is below physiological oxygen concentration, then, less than 9% (Figure 1). This hypoxia directly induces the overexpression of hypoxia-inducible factors (HIFs). These hypoxia-related biomarkers were in past studies found to be frequently associated with the worst OS in many cancers, including OTS. HIF-1α hyperexpression was significantly linked with metastatic disease, poor prognosis and outcome in OTS, supporting its probable role at diagnosis and during progression [15,16,17,18]. Usually, hypoxia confers a more aggressive phenotype by activating a cascade of molecular events partly mediated and regulated by HIFs. These also play an essential role in immunological responses and are considered as crucial physiological regulators of homeostasis, vascularization and anaerobic metabolism. So, the hypoxia-specific tumor microenvironment might be of importance during OTS oncogenesis, progression and during their treatment. Therefore, we try to decipher the normoxic/hypoxic pathways in normal tissues, bones and osteosarcomas to understand their role and to estimate their druggability in OTS. To study the efficacy of drugs inhibiting hypoxic biomarkers, we now need specific in vitro models closely mimicking the tumor and its intra-tumor hypoxia.

## 2. Biomarkers Related to Hypoxia Regulation in Normal Tissues

**Figure 2:**
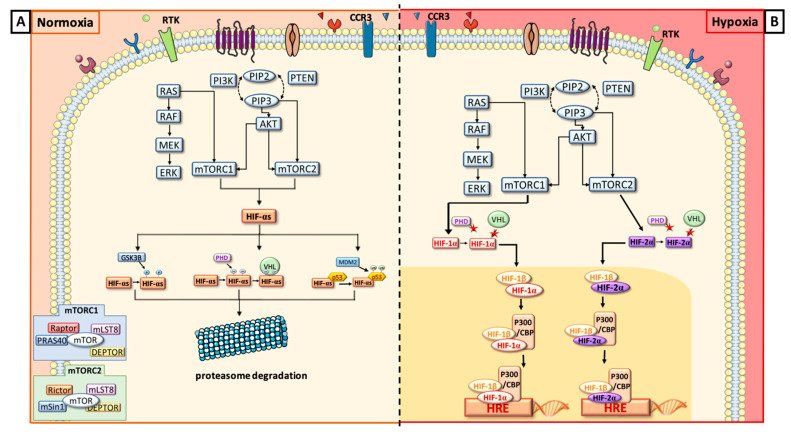
Schematic description of the biomarkers involved in normoxia (**A**) and hypoxia (**B**) and their interplay.

In response to a decrease in oxygen level, HIFs are induced and will rapidly after induction regulate the downstream- and upstream-related pathways (descriptions in Figure 1 and Figure 2). These transcription factors are heterodimeric DNA-binding complexes with a basic helix-loop-helix-PAS domain and comprise one α subunit (HIF-1α, HIF-2α or HIF-3α) and the paired β subunit. Knowledge is very limited for the role of HIF-3α, but HIF-1α and HIF-2α are frequently described in hypoxia regulation [19]. The aryl hydrocarbon nuclear translocator (ARNT), the β unit, is constitutively expressed in the cell nucleus and its rate is constant and independent of oxygen tension. The α subunits are in normoxic conditions hydroxylated at their proline residues (PHD) by HIF prolyl-hydroxylases to be, thereafter, ubiquitinated by the pVHL (protein Von Hippel-Lindau) ubiquitin ligase complex and degraded by the proteasome (Figure 2A). In hypoxia, HIF prolyl-hydroxylases are inhibited and HIF-α subunits are stabilized to translocate into the nucleus, where they heterodimerize with the β unit (Figure 2B). The heterocomplex composed of the HIF-α and HIF-β will bind the hypoxia response element (HRE) with the coactivators CBP and p300 and recognize the promoters of a large number of genes to enhance their transcription [20,21,22,23]. Physioxia (Figure 1) seems to be part of these hypoxia-related pathways and is associated with a modulation of stress signals that can induce a balance between proteasomal degradation of HIFs-α and their stabilization [24]. No clear-cut threshold separates physioxia from hypoxia nor normoxia.

Globally, HIFs are regulated by their upstream signaling cascade in all oxygen tensions (Figure 2A,B). When PI3K/AKT/mTOR or RAS/RAF/MEK/ERK pathways are upregulated, HIF-α mRNA transcriptions and protein translations are promoted [25,26,27,28]. The distinct roles of HIF-1α and HIF-2α cover the regulation of cell differentiation and promotion of the tumor cell resistance and invasion. HIF-1α is known to activate acutely more than one hundred genes [19] in combination with PI3K/AKT/mTOR [29,30], RAS/MAPK [21,30] and NF-kB pathways [31]. These molecular cascades will activate cell proliferation and oncogenic features, cell migration and invasion [32]. Locally or during metastatic processes, HIF-1α will induce a neoangiogenesis with VEGF/VEGFR (vascular endothelial growth factor and receptor) cascade and an anaerobic glycolytic switch, pushing cells in a specific phenotype in case of acute hypoxia. It will also play a role in epithelial–mesenchymal transition, as well as in the tumor cell resistance to therapies through p53 or upstream signaling [16,20,30,33,34,35]. HIF-2α regulates, like its HIF-1α counterpart, many physiological functions, such as local or metastatic neoangiogenesis, cell proliferation, and migration [23,30,33,36]. For the metabolism, HIF-2α seems to promote an alternative metabolic switch like phospholipid or amino acid metabolisms [37]. Through its specific roles, HIF-2α mostly drives the response to chronic hypoxia, maintaining immature tumor cells [23,32,33,38,39]. Both HIFs are modulated and combined to adapt normal and cancer cells to oxygen variations and tensions. They have probable compensatory activities because, when HIF-1α is underexpressed, HIF-2α can in different conditions increase its expression. Nevertheless, this modulation is probably in a tissue dependency manner [11,19,23,24,40].

Just upstream to HIFs (Figure 1 and Figure 2), there is the mammalian target of rapamycin (mTOR), a serine/threonine kinase, which is represented by two subunits: mTORC1 and mTORC2. They have several common components: mTOR kinase, which is the central catalytic component, mLST8, a scaffolding protein, and Deptor, a regulatory subunit. mTORC1 is associated with Raptor, a scaffolding protein necessary to stabilize its subcellular localization and with PRAS40, inhibiting mTORC1 activity in absence of growth factor. mTORC2 is composed of Rictor and mSin1, a negative regulator. Rictor and Raptor have similar functions [41,42]. To simplify, usually, mTORC1 upregulates HIF-1α via the S6 ribosomal protein (S6K), whereas mTORC2 directly activates HIF-2α [25,32,33].

Upstream to mTOR, we have two signaling cascades, both stimulated by tyrosine kinase receptor phosphorylation: PI3K/AKT and RAS/ERK/MAPK. The production of phosphatidylinositol (3,4,5)-triphosphate (PIP3) will directly activate mTORC2, which phosphorylates Akt on ser473. mTORC1 is activated by PI3K/AKT in two different ways: either by phosphorylation of PRAS40 or by the inhibition of tuberous sclerosis complex 2 (TSC2) [43,44]. RAS indirectly activates mTORC1 and ERK HIF-1α through S6K [45].

In parallel to these cascades, where oxygen variations are predominant drivers [46], there are two oxygen-independent conditions where HIFs can be regulated by other mechanisms than the pVHL-induced proteasomal process. Indeed, HIF-1α will also undergo proteasomal degradation, after it binds p53 protein leading to ubiquitination by MDM2 (Murine Double Minute 2), another ubiquitin ligase. Another mechanism of HIF-1α degradation is driven by glycogen synthase kinase 3b (GSK3B). In fact, GSK3B is known to phosphorylate HIF-1α and then enhance its degradation [47]. Furthermore, a specific mechanism, called pseudo-hypoxia (Figure 1), is also described for HIF-α induction in normoxic conditions. It is a compromised cellular capacity of utilizing oxygen due to decreased levels of nicotinamide adenine dinucleotide (NAD), which can cause the accumulation of NADH with the occurrence of NADH/NAD redox imbalance. These metabolic modifications might induce HIF production or stabilization mainly through stress signals and ROS accumulations [19].

## 3. Normal Bone and Hypoxia: Involvement in Osseous Production and Formation and Osteoclast-Mediated Bone Resorption

Bone and its associated marrow microenvironment offer access to growth factors, cytokines, blood supply and tumor-supportive cells including macrophages, T cells and stromal cells. The osteoblasts and the osteoclasts play a pivotal role in skeletal development and remodeling. Bone can form through two different mechanisms: intramembranous or endochondral ossification. The intramembranous bone formation is dedicated to flat bones. It develops from mesenchymal cells that directly differentiate into osteoblasts for the skull. In other flat skeletal locations, it derives from a chondrocyte anlage that is replaced by bone [48,49,50]. For the endochondral bone development, we have three steps where physioxia and hypoxia play major roles. The first step is the condensation of mesenchymal cells, which will next differentiate into chondrocytes and, at the end, generate the growth plates. In the growth plates, chondrocytes are highly proliferative and will form columnar layers, where, in the most distal part, cells will stop proliferating, exit the cell cycle and differentiate into hypertrophic chondrocytes associated with mineralization [50,51]. The growth plate is a unique mesenchymal tissue with avascular and hypoxic regions [52]. To overpass this challenging microenvironment, the chondrocytes need HIF-1α expression and production of VEGF-A to induce the angiogenic switch and be able to replace cartilage by bone [51]. This overexpression is associated with pVHL expression, that is modulated by HIF-1α, and the stabilization of HIF-2α, which is balancing HIF-1α action [53]. In fact, the HIF signaling pathway has a critical role in regulating both the osteoblastic and the vascular niches during the endochondral process. HIF-1α is considered as a positive regulator of bone formation, as well as osteoblast number and activity. It stimulates non-oxidative glycolysis in osteoblasts and can delay osteoclastogenesis, favoring the senescence of bone macrophages [51]. So, HIF-1α will play a role of regulator of osteoclast-mediated bone resorption, but with little effect on osteoclast differentiation itself [54]. It stimulates the expression of cytokines that might regulate the differentiation process and increases both the glycolytic and mitochondrial metabolic rate to provide an adaptive support to the macrophages during bone resorption [35,55]. By contrast HIF-2α can, then, be considered as a negative regulator of bone mass accrual with a direct action on osteoblast lineage [51]. In compensation, MIF (macrophage migration inhibitory factor) seems to regulate HIF-1α activity in a p53-dependant manner. This physiological oxygen tension and pathological hypoxia during cancer processes might in this context of hypoxic growth plate be a good site to favor tumor cell homing and initiation, as well as tumor expansion. It can also explain the tight interactions between malignant osteoblast and its immune environment, as well as blood vessel formation and the macrophages infiltrating tumor microenvironment.

## 4. Presence of Hypoxic Biomarkers in Osteosarcomas Is Related to Progression and Resistance to Treatment

Hypoxia seems to be an important key in OTS local and distant environments. As described above, the local environment of growth plates is conducive to favor variations in oxygen levels and is typically the location where osteosarcoma cell arise. The hypoxic biomarkers seem to be modulated during osteosarcoma initiation and progression. Correlations have been established in numerous publications between OTS poor prognosis and those biomarkers including HIFs, mTOR or CA IX (Carbonic Anhydrase IX) [16,17,18,47,56,57,58]. They are underlining the dominant driving force of hypoxia for OTS cancer progression, drug resistance and metastatic propensity. When looking at the material used in studies, the proof of concept for hypoxia in osteosarcomas was made in in vitro or in vivo preclinical models, as well as in tumor collections. However, constantly, HIF-1α was the central hypoxia-related marker involved in OTS and is frequently hyper-expressed in locally aggressive and metastatic OTS [17,18,19,58,59]. It is associated with GLUT-1 (GLucose Transporter 1), CA IX or VEGF/VEGFR overexpression [57,58,59], explaining a global enhancement of the hypoxia pathways from the membrane to the nucleus and the increase of intra-tumor microvessel density [20]. HIF-1α was, then, mostly described as a major driver of tumor microenvironment modulation. HIF-2α was less studied in OTS than its homologues, but seems to be implicated in OTS cell proliferation and apoptosis and promotes OTS stemness features [17,36,38,56,60,61]. So, HIF-2α was mostly described as a major driver in OTS cells with specific metabolic switch [40]. mTOR was frequently involved in direct links with autophagic processes and was associated with Pi3K/AKT upstream signaling stimulated by different tyrosine kinase receptors [62,63,64], combining an OTS cell and tumor microenvironment effect. Nevertheless, it is for instance not so clear how all those biomarkers specifically interact during OTS progression and metastatic propension, as hypoxia is present since the OTS cell initiation. A balanced HIF-1α/HIF-2α, as well as a variation of expression of both markers, are now more precisely described to explain the intermittent role of hypoxia signaling pathways in many cancer types, but must be more studied in OTS [33,47].

Recent insights also associate a global intra-tumor hypoxia to elevated genomic instability in cancer cells, including osteosarcomas, where a high level of chromosomal breakage and chromothripsis are usually observed [15,65]. This genomic instability and complexity is linked to a poor outcome and is frequently correlated in such tumor types with a high dysregulation of microRNAs subsequent to hypoxia. In OTS, it is miRNA-133a that was probably shown to be part of chromosomal deregulation and osteosarcoma progression [66].

The epithelial–mesenchymal transition (EMT) is a mechanism also playing a role in OTS proliferation and cell invasion. One central signaling pathway was particularly deciphered in OTS EMT process, which is Wnt/β-catenin signaling. Surprisingly, studies more often describe a balance of down- and up-regulation to explain the role of the Wnt/βcatenin pathway. In fact, it is up-regulated to favor cell proliferation, colony formation and migration [67], but it is down-regulated by hypoxia and, then, promotes cell resistance to chemotherapies through stemness properties and MDR (MultiDrug Resistance) induction [68]. For these treatment resistances, several other studies have focused on hypoxia as a way for OTS cells to adapt to their new environment and induce neoangiogenesis to favor cell proliferation and tumor growth, avoiding autophagic and apoptotic response to therapies [8,11,33,38,68,69]. They confirmed that MDR phenotype is reactivated during oxygen hypoxic tension [70]. Another process was the inhibition by PI3K/AKT and HIF-1α of MAX dimerization protein 1 (Mxdm1), a member of the Myc/Mxd/Max family, to notably overpass cisplatin DNA toxicity in OTS cells [70,71]. Finally, the overproduction of reactive oxygen species (ROS) interplays with OTS hypoxia and the AMPK signaling pathway, as well as autophagy and another hypoxic biomarker [71,72], to promote treatment resistance for chemotherapeutic strategies and for irradiation [57].

## 5. Immune Response, Hypoxia and Osteosarcoma Cells

The OTS microenvironment is surprisingly characterized by specific immune infiltrates in which mostly macrophages and osteoclasts are present. T cell response, able to fight against the tumor cells, is suppressed in OTS leading to a T-cell exhaustion. It induces immune tolerance and prevents excessive immune responses leading to tumor growth, drug resistance and/or metastatic spread [73,74,75]. Besides the fact that programmed death receptor-1 (PD-1) and its ligand PD-L1 seem to be interesting cancer targets, PD-L1 is absent in the OTS primary tumor. The PD-1/PD-L1 expression is described mostly in metastatic locations and is linked to a poorer outcome [76,77]. Indoleamine 2,3-dioxygenase 1 (IDO1) is another suppressive protein, which is also minimally expressed as PD-1 and only in metastatic disease [77]. Nevertheless, this T-cell immunity is rare in OTS, explaining the fact that predominant immune system balance is macrophage based [74,77,78,79,80]. Hypoxia is known to attract myeloid-derived suppressor cells and tumor-associated macrophages (TAMs).

In the innate immune response, osteoclasts favor metastatic OTS spread with local environment destruction. A balance between M1, considered as pro-inflammatory and anti-tumoral macrophages, and M2, considered as pro-tumoral macrophages, is frequently described in OTS to explain the response to macrophage modulators, as well as the capacity to have local or distant progression. This M2 polarization phenotype and specific TAM infiltration change during OTS tumor growth represents a whole dynamic process precisely regulated by hypoxia [59,79,81,82]. Hypoxia and especially the PI3K/AKT/mTOR upstream pathway are known to regulate this osteoclastogenesis phenomenon [82,83], but also the immune system through cell surface protein modulations. This balanced M1/M2 phenotype in OTS is complex, but growing evidence suggests that a high density of M2 TAM is associated with OTS primary and metastatic locations. The TAM recruitment in the OTS microenvironment is hypoxia-dependent and in direct link with several chemokines and their receptors.

In fact, M1 macrophages, which are activated by interferon γ or lipopolysaccharide, exhibit anti-tumor properties through the production of pro-inflammatory cytokines (interleukin-1β and interleukin-6) and inducible factors against pathogens such as the tumor necrosis factor α (TNF-α) and the nitric oxide synthase (INOs). HIF-1α upregulation usually stimulates the amino acid metabolism and promotes nitric oxide synthase activity. When suppressing the M1 macrophage activity, as in the recent OS2006 therapeutic protocol, it can promote a poorer outcome in metastatic disease [81]. The presence of the M1 subtype was therefore linked to a better OTS patient outcome [78,79,80]. M2 macrophages, instead, are activated by anti-inflammatory cytokines (interleukine-4 and interleukine-10) and the PI3K/AKT/mTOR pathway, and exert immunosuppressive effects associated with enhanced angiogenesis and tumor progression, pushing OTS cells in stemness status [78,82,83]. In fact, they are able to suppress T cell proliferation [84], favor angiogenesis through VEGF and angiopoietin signaling, and enhance cancer stem cell properties by upregulating CD133+ cells [34].

Additional effects of hypoxia reinforce this M2 pro-tumoral phenotype. In fact, hypoxia can also decrease the expression of cell surface MHC class I-related chain molecules A (MICA) and prevents the immune cells degrading the tumor cell via a HIF-1α-dependent pathway linked to increased expression of metalloproteinase (MMP) [77]. Macrophage migration inhibitory factor (MIF) interplays with HIF-1α protein overexpression and stabilization [50] for the promotion of OTS tumorigenesis, whereas osteoclast activity is enhanced by the hypoxia-induced ANGPTL4 (angiopoietin-like 4) overexpression [8].

Recent studies have linked hypoxia and immunomodulation scores in OTS [77,85]. This score also supports the idea that hypoxia-driven immunity is associated with glycolytic metabolic switch, collagen biosynthesis and redox regulation. Globally, HIFs and mTOR pathway exert a tumor-promoting effect by intra-tumor immunosuppression.

## 6. HIFs Targeting in OTS

**Figure 3:**
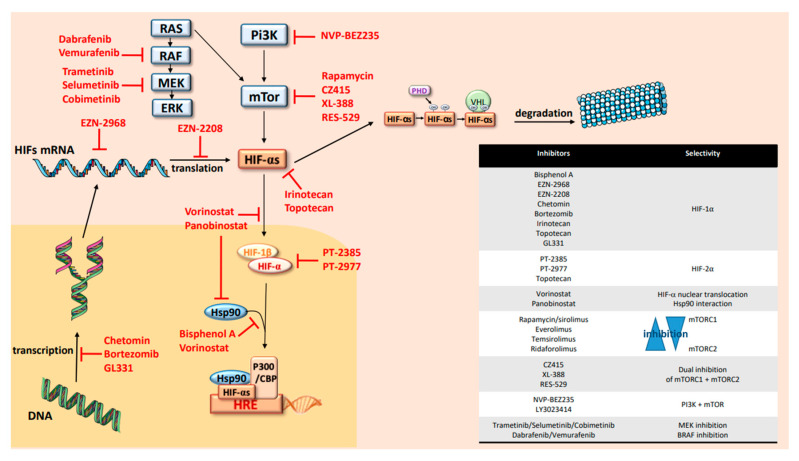
Hypoxia cascade targeting.

All these findings point out the crucial role of HIFs in osteosarcoma initiation, progression and immune dynamic modulation. They suggest that HIFs and their upstream and downstream pathways might be key targets in OTS treatments. Many hypoxia inhibitors exist to stop directly or indirectly the hypoxic pathways and now are progressively used in clinical trials. When focusing on HIFs, several levels of inhibition can be listed and are summarized in Figure 3. In fact, a growing number of molecules have been demonstrated to inhibit HIFs by reducing mRNA or protein levels, DNA-binding activity or the trans-activation of some HIFs related genes [85]. They can also block HIFs/HIF-1β dimerization. The synthesis of HIF-1α is strictly related to mTOR activity. As described above, the continuous activation of PI3K/AKT and RAS/MAPK signals determines the increase of mTOR activity and the consecutive activation of HIFs [26,27]. Thus, inhibitors of those biomarkers are also able to inhibit HIF activity and reduce their impact on OTS cell adaptation.

HIFs, as transcription factors, have been considered undruggable for a long time [40]. To date, no specific inhibitors of HIF-1α have been brought to the market, but the use of indirect or partial inhibitors are currently increasing in trials and recently in OTS protocols. Oppositely, recent direct inhibitors of HIF-2α have been developed and commercialized, such as PT2385 or PT2977, but are not used directly in OTS and only preclinical data show that HIF-2α targeting can attenuate the proliferation, migration and invasion of OTS cells [61,85,86]. Those specific inhibitors of HIF-2α destabilize heterodimerization of HIF-2α with ARNT, leading to inhibition of target genes and tumor regression. These treatments were used recently in clinical trials for patients with clear cell renal cell carcinoma, where the majority of patients had a partial response or a stable disease associated with a good tolerance [61,85,86]. The HIF-1α inhibitors are mostly decreasing HIF-1α expression with a proven mRNA downregulation in the OTS preclinical models or in cancer trials [85,86,87,88,89,90]. So, molecules like EZN-2968 or EZN-2208 and topoisomerase I (irinotecan) and II (topotecan and GL331) are frequently used for this purpose. A small number of pediatric studies have proposed these molecules, which were well tolerated and efficient in hypoxic tumors like neuroblastomas. Only topotecan seems to have a dual effect of HIF-1α/HIF-2α. Histone DeACcetylase (HDAC) inhibitors, like vorinostat or panobinostat, can also block HIF-1α nuclear translocation via direct acetylation of its associated chaperone, heat shock protein 90 (Hsp90) [90]. Artificially, the same mechanism of inhibition was also shown with Bisphenol A that is not used in clinics [91]. Another way to stop HIF transcription is to interrupt interaction between HIF-1α and its coactivator p300, which results in a mitigation of hypoxia-inducible transcription [92]. Proteasome inhibitors like bortezomib might also interact with HIF transcription and can be used efficiently alone or in combination with other targeted therapies of the mTOR/HIF pathway [93,94]. Upstream to HIFs, PI3K/AKT, RAS/ERK/MAPK and mTOR are also good candidates for targeted treatments using specific mTORC1 and/or mTORC2 inhibitors or combining mTOR and PI3K antagonists [62,72,95,96]. In parallel, RAS/BRAF/MEK/ERK mitogen-activated protein kinase cascade is known to be involved in OTS. It integrates signals from cell surface receptors to activate ERK and such upregulation can be also targeted by MEK or BRAF inhibitors. In fact, multiple selective, orally available, non–ATP-competitive small-molecules are now available in clinics to block MEK1 and MEK2 (MAPK kinase) proteins and might be administered in new OTS trials. They might be proposed in a single drug approach or combined with BRAF inhibitors.

Finally, when inhibiting mechanisms induced by hypoxia, efficient drugs should promote tumor growth and cell proliferation arrest through an up-regulation of caspase-3. These molecules might also attenuate tumor angiogenesis leading to efficacy antitumor strategies and reduce usually radio- and chemoresistance of human OS cells in hypoxic conditions. Nevertheless, frequent oncogenic addictions are described due to an AKT reinduction after mTOR or HIFs inhibition, leading to a selective resistance to these inhibitors and conducting the proposal of more and more combinations in phase I and II trials [62,72,94].

When looking ar osteosarcoma trials on the https://clinicaltrials.gov website, fewer than 15 currently opened protocols are using such strategies with HIFs inhibitors such as irinotecan, mTor inhibitors (ridaforolimus, everolimus, sirolimus) or AKT/Pi3K inhibition with dual mTOR/PI3K targeting. More recently, other therapies independently from HIFs, such as the CA IX inhibitors or strategies targeting metabolic OTS cell vulnerabilities, seem to be effective in in vitro and in vivo models with an increase of cytotoxicity on OTS cells [97].

The future of all those therapies’ proposals is to combine treatments in order to increase the effects and decrease therapeutic resistance.

## 7. In Vitro and In Vivo Models to Recreate OTS Hypoxic Microenvironment

**Figure 4:**
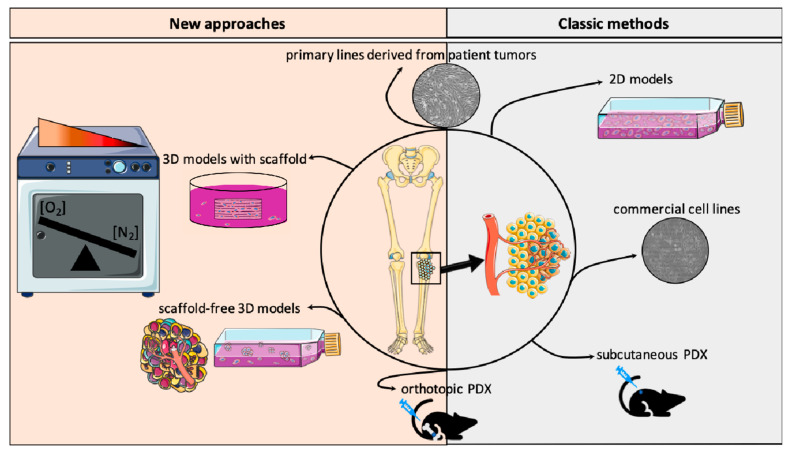
In vitro and in vivo models recreating osteosarcoma and hypoxic environment.

To study hypoxia features and test druggability of those biomarkers, a recent effort was made to develop appropriate models to mimic closely both OTS patient cells and the tumor microenvironment (Figure 4). First, the OTS cells themselves are now mostly patient-derived cell lines based on diagnostic tumor or relapse tissue specimens. In fact, for several years, there have been commercial cell lines allowing the study of OTS. In order to get as close as possible to the physiological conditions, it is important to develop cell models able to recreate the OTS behavior and heterogeneity and the tumor microenvironment, where several supporting stromal cell subtypes are present [98]. For this purpose, more and more 3D-based approaches have been developed and offer advantages over the monolayer-based cultures (Figure 4).

The evolution of 3D OTS culture systems now takes into account the engineered osteoid matrix, as well as oxygen concentrations, angiogenic cells or intrinsic immune components (e.g., macrophages or lymphocytes). They overlap the tumor spheroids to the complex organoid-derived models, where the endogenous infiltrating immune stroma is automatically present. In sphere culture, co-culture with angiogenic cells like HUVEC, as well as macrophages, can partially replace the stromal or immune subtypes observed in initial patient tumors. For the bone environment, one major part is the osseous matrix, which can be mimicked by natural (e.g., collagen, Matrigel) or synthetic scaffolds (e.g., hydrogels) [99]. For hypoxia features and oxygen variations, hypoxia chambers are the most accurate culture environment, where deep intra-tumor hypoxia might be approximate (Figure 4).

Patient tumor cells can also be injected subcutaneously or orthotopically in mouse models [100]. Unfortunately, in these models, metastatic modelization is variable and probably more frequently accessible during orthotopic injections. To facilitate tumor cell dissemination and metastatic spread, some publications describe femoral artery ligation close to the orthotopic injection to promote a hypoxic environment and enhance the probability of success in obtaining pulmonary metastases.

The final goal of all those models is to obtain more representative results after drug testing and allow a rapid translation of those preclinical data into new innovative therapeutic trials for patients.

## 8. Conclusions

Development of innovative therapies for worst outcome OTS is an unmet medical need. The extended knowledge in hypoxia-driven OTS development offers understanding of crucial key points of OTS progression and interaction with its immune environment. It also helps to support the development of personalized hypoxia-targeting trials in OTS, as drugs are available and might be used in combination. Nevertheless, to improve targeting hypoxic biomarkers, a number of challenges need to be addressed in specific 3D preclinical models integrating hypoxia, bone matrix, patient-derived OTS cells and the endogenous immune-infiltrating macrophages.
